# Optimal human papillomavirus vaccination strategies in the context of vaccine supply constraints in 100 countries

**DOI:** 10.1016/j.eclinm.2024.102735

**Published:** 2024-07-18

**Authors:** Kiesha Prem, Tania Cernuschi, Stefano Malvolti, Marc Brisson, Mark Jit

**Affiliations:** a Department of Infectious Disease Epidemiology, London School of Hygiene & Tropical Medicine, Keppel Street, London, WC1E 7HT, United Kingdom; b Saw Swee Hock School of Public Health, National University of Singapore, 12 Science Drive 2, #10-01, 117549, Singapore; c World Health Organization, 1202, Geneva, Switzerland; d MMGH Consulting, 8049, Zurich, Switzerland; e Centre de Recherche du CHU de Québec - Université Laval, Québec, QC, Canada; f Department of Social and Preventive Medicine, Université Laval, Québec, QC, Canada; g MRC Centre for Global Infectious Disease Analysis, Department of Infectious Disease Epidemiology, Imperial College London, London, UK; h School of Public Health, University of Hong Kong, Patrick Manson Building, 7 Sassoon Road, Hong Kong SAR, China

**Keywords:** Human papillomavirus, Vaccination, Cervical cancer, Vaccine allocation, Modelling

## Abstract

**Background:**

Countries are recommended to immunise adolescent girls routinely with one or two doses of human papillomavirus (HPV) vaccines to eliminate cervical cancer as a public health problem. With most existing vaccine doses absorbed by countries (mostly high-income) with existing HPV vaccination programmes, limited supply has been left for new country introductions until 2022; many of those, low- and middle-income countries with higher mortality. Several vaccination strategies were considered by the Strategic Advisory Group of Experts on Immunization to allow more countries to introduce vaccination despite constrained supplies.

**Methods:**

We examined the impact of nine strategies for allocating limited vaccine doses to 100 pre-introduction countries from 2020 to 2030. Two algorithms were used to optimise the total number of cancer deaths that can be averted worldwide by a limited number of doses (knapsack and decreasing order of country-specific mortality rates), and an unoptimised algorithm (decreasing order of Human Development Index) were used.

**Findings:**

Routinely vaccinating 14-year-old girls with either one or two doses and switching to a routine 9-year-old programme when supply is no longer constrained could prevent the most cervical cancer deaths, regardless of allocation algorithm. The unoptimised allocation averts fewer deaths because it allocates first to higher-income countries, usually with lower cervical cancer mortality.

**Interpretation:**

To optimise the deaths averted through vaccination when supply is limited, it is important to prioritise high-burden countries and vaccinating older girls first.

**Funding:**

10.13039/100004423WHO, 10.13039/100000865Bill & Melinda Gates Foundation.

**Research in context**

**Evidence before this study**

We searched PubMed for studies published from 2012 with the terms “((human papillomavirus) OR HPV) AND (vaccine OR vaccination) AND (constraint OR limited OR constrained) AND (supply OR dose)” and identified 211 results. Ten published articles highlighted the HPV vaccine supply constraints, and only one published study evaluated the impact of alternative vaccination strategies to maximise benefits in cervical cancer prevention in four low-income and middle-income countries under vaccine supply constraints. However, to our knowledge, no published article has explored or modelled the impact of alternative vaccination strategies on cervical cancer prevention under supply constraints across all pre-introduction countries.

**Added value of this study**

This study presented the first evidence on the optimal use of scarce vaccine doses across 100 pre-introduction (in 2020) countries and evaluated the impact of nine vaccination strategies on cervical cancer prevention. Prioritising the vaccination of older girls who are close to ageing out of vaccine eligibility in routine programmes would be the most efficient approach, and would help maximise the number of deaths prevented with the available doses.

**Implications of all the available evidence**

When vaccine supplies are limited, dose-sparing strategies, such as giving just one dose or vaccinating older girls first, have the potential to prevent more deaths from cervical cancer. Failure to prioritise high-burden countries for HPV vaccination may result in significant human costs. Findings were presented to SAGE to inform recommendations about extended interval and single-dose schedules when there is a limited global supply of HPV vaccines.

## Introduction

Persistent infection with a high-risk human papillomavirus (HPV) type is a necessary cause of cervical cancer and is preventable with HPV vaccination.^1^ Vaccination of adolescent girls is a cost-effective public health measure against cervical cancer.^2^ As of November 2023, more than 140 countries have introduced the HPV vaccine into their national schedules.^3^^,^^4^ The World Health Organization (WHO) recommends that routine HPV vaccination programmes target girls between 9 and 14 years of age.^5^ In the first year of HPV vaccine introduction, countries may also implement multiple age-cohort (MAC) vaccinations, vaccinating multiple cohorts of girls that are too old to be reached by routine programmes.^6^

In 2020, WHO launched a global strategy to accelerate the elimination of cervical cancer as a public health problem. Vaccination is one of the three key pillars of a comprehensive strategy to eliminate cervical cancer as a public health issue. This initiative envisions that all countries achieve 90% age-eligible female HPV vaccine coverage by 2030.^2^ The other two pillars focus on screening and treating pre-cancerous lesions, treating cervical cancer cases, and providing palliative care.^2^ Although WHO recommends all countries to immunise adolescent girls with one or two doses routinely, many low- and lower-middle-income countries have not.^4^^,^^7^, ^8^, ^9^ Initially, low uptake was driven by financial or programmatic barriers of delivering two doses outside the established infant immunisation programme.^10^^,^^11^ To narrow the equity gap, Gavi, the Vaccine Alliance, provides support for HPV vaccine introduction in many low- and lower-middle-income countries. WHO's recent shift from recommending two doses to recommending either one or two doses of vaccine may further expand the number of countries able to introduce vaccination.

Unfortunately, in the late 2010s, HPV vaccine implementation in low- and lower-middle-income countries was further delayed due to continuing constraints in vaccine supply.^12^^,^^13^ The global HPV vaccine supply shortage was precipitated by a wave of middle-income country introductions as well as a move to gender-neutral vaccination in high-income countries.^12^^,^^14^^,^^15^ This stymied introduction opportunities for the remaining countries. Of the 100 countries that were yet to introduce a national HPV immunisation programme in May 2020, 60 were low- and lower-middle-income countries and 24 were upper-middle-income countries. Many low- and middle-income countries introducing the vaccine had to postpone their introduction and plans for MAC vaccinations.^16^ In the early 2020s, while vaccine supply was still constrained,^12^^,^^14^^,^^15^ the constraints were partially eased because of a decrease in demand resulting from the disruption in vaccination programmes during the COVID-19 pandemic.^13^^,^^17^ Additionally, the COVID-19 pandemic and reactive lockdowns affected the short-term supply outlook by delaying introduction of new HPV vaccine candidates in clinical development.^18^^,^^19^ On the demand side, diversion of workforces to address COVID-19 outbreaks as well as pandemic-related school closures have caused interruptions to existing vaccination programmes as well as delays to vaccine introductions.^17^^,^^19^^,^^20^ While the disruption in demand, as well as more recent increases in vaccine supply led to an increase in the availability of vaccines, as countries introduce or resume vaccination, campaigns targeting missed cohorts needed still to be phased to ensure equitable use of the vaccines.^18^

Together with their partners, WHO and Gavi are working with manufacturers to ensure ramp-up of production capacity to meet the global demand.^15^ Moreover, to ensure sufficient vaccine supply for all the remaining countries to introduce HPV into their national immunisation schedule by 2030, several vaccination strategies were assessed and discussed during the meetings of the WHO Strategic Advisory Group of Experts (SAGE) on Immunization in 2019^16^ and 2021.^18^ Out of these strategies, SAGE recommended an extended two-dose vaccine schedule to allow more countries to introduce the HPV vaccine despite constrained supplies, the modelling results from this study also helped inform SAGE's decision to recommend a single-dose schedule. Here we present the modelling evidence that informed this decision, as well as updates to account for likely changes in supply projections following the COVID-19 pandemic.^19^ Using mathematical modelling, we explore the impact of the nine vaccination strategies on cervical cancer cases, deaths and HPV vaccine introductions between 2020 and 2030^12^^,^^14^ for 100 countries in the context of the vaccine supply constraints projected in 2019 and adjusted for the impact of the pandemic.

## Methods

### Global HPV vaccine supply

**Fig. 1:**
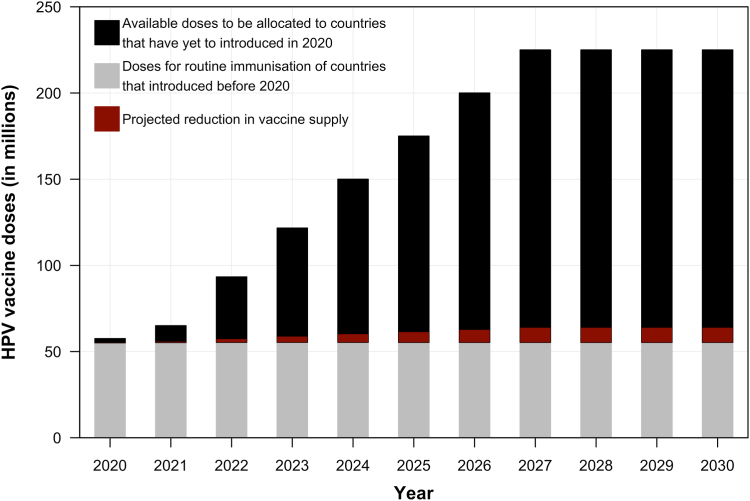
**Projected global supply of HPV vaccines in 2020**–**2030.** The grey area represents the dose required for routine immunisation for the years 2020–2030 in the countries that have introduced or partially introduced HPV vaccines in the national immunisation programme before 2020. The black area represents the dose available for routine immunisation in the 100 countries that have yet to introduce HPV vaccines in the national immunisation programme before 2020. The red area represents the potential impact of COVID-19 on HPV vaccine supply (e.g., 5% reduction in projected supplies as depicted in the figure).

**Fig. 2:**
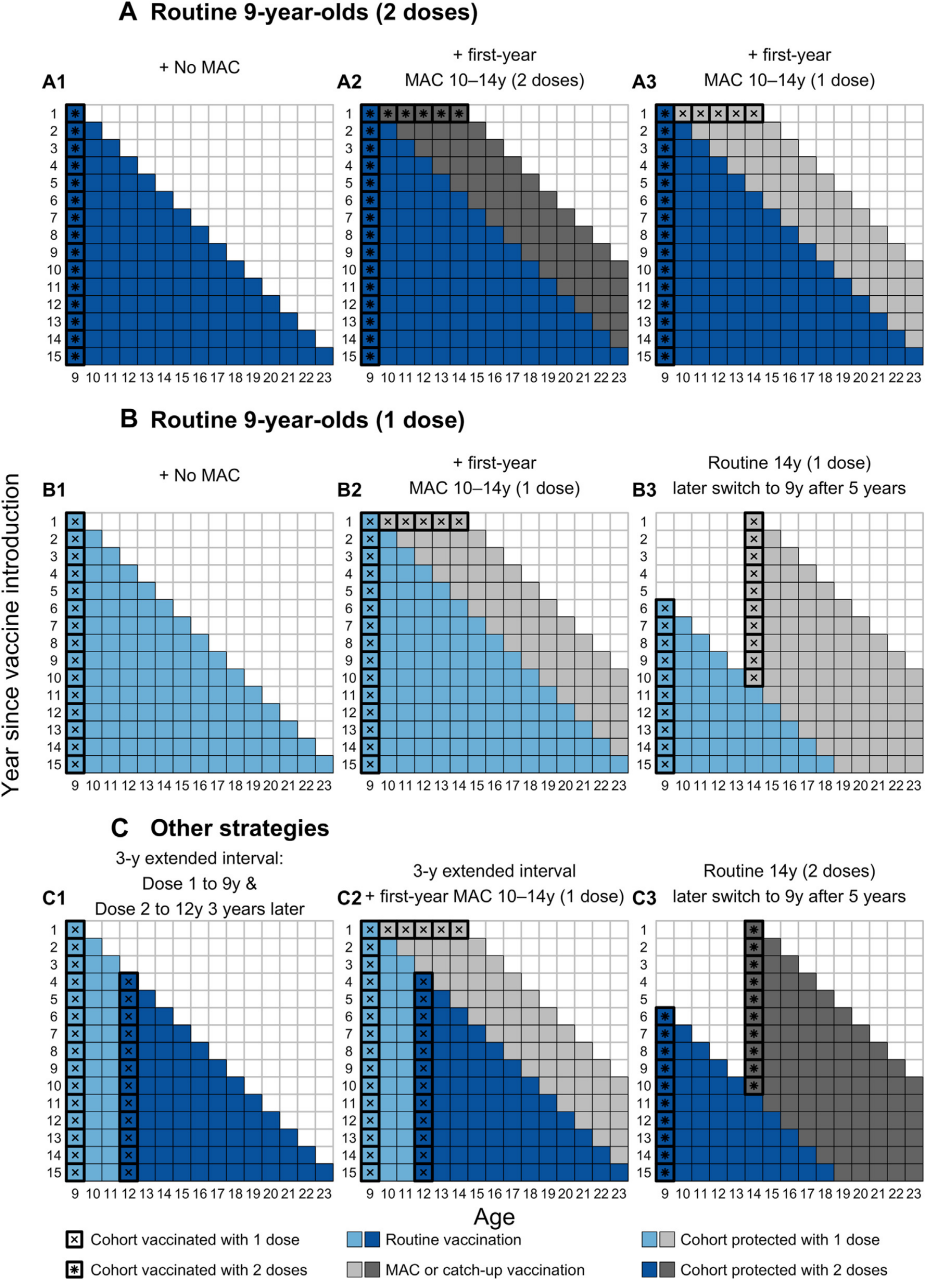
**Vaccination strategies.** The Strategic Advisory Group of Experts on Immunization considered several human papillomavirus vaccination strategies which allowed more countries to introduce vaccination despite constrained vaccine supplies.

The global supply of available HPV vaccine doses for each year in 2020–2030 was estimated based on the findings of WHO's HPV vaccine market study from 2019.^12^^,^^14^ We then assumed that the COVID-19 pandemic further reduced vaccine supply by 5% compared to 2019 projections in the base case.^14^^,^^18^^,^^19^ We varied this reduction between 0% (i.e., no net impact) and 10% in sensitivity analyses. The initial distribution of global vaccine supply was first allocated to countries with established national routine HPV immunisation programmes initiated before 2020 (depicted by the grey bars in Fig. 1). The remaining vaccines were then distributed according to one of nine vaccination strategies, which cover strategies with and without MAC or single-year catch-up, one- and two-dose and extended interval schedules (more details in Fig. 2 and Supplementary Table S1).

### Statistical analysis

#### HPV vaccine impact modelling

Papillomavirus Rapid Interface for Modelling and Economics (PRIME) is a WHO-supported model of HPV vaccination that uses proportional impact to estimate the cost-effectiveness of the HPV vaccination.^21^^,^^22^ We used PRIME to estimate the number of cervical cancer deaths averted under the different HPV vaccination strategies compared to a scenario with no vaccination (lost health impact). PRIME provides conservative estimates of the vaccine impact as the static model does not account for herd effects and cross-protection against non-vaccine HPV types. It adjusts for lower vaccine protection in vaccinees who have sexually debuted before vaccination.

We modelled vaccination with the 2-valent vaccine and assumed vaccine coverage to be 90%. We only considered cervical cancer deaths caused by HPV 16 and 18, which the 2-valent HPV vaccine could prevent.^23^ Cervical cancer mortality data were sourced from the Global Cancer Incidence, Mortality and Prevalence (GLOBOCAN) database, providing data on age-specific cervical cancer incidence, prevalence, and mortality among females (https://gco.iarc.fr/). Two doses of the HPV vaccine were assumed to have a lifelong efficacy of 100% against vaccine-preventable deaths. While most trials and post-trial analyses have not shown a difference between the efficacy of one and two doses, we assumed that one dose of the vaccine has lifelong 90% efficacy (and 95% in a sensitivity analysis). This assumption around the efficacy of the one-dose regimen is substantiated by the KENSHE trial, which found that the vaccine efficacy of the one dose of HPV vaccines is similar to multidose regimens in preventing incident persistent oncogenic HPV infection.^24^ In this analysis, we also assumed the duration of protection from one-dose vaccination to be lifelong but at lower vaccine take than two-dose. A total of 100 runs were simulated from the parameter sets to capture uncertainty in the incidence and mortality estimates and the proportion of cancers prevented by vaccination.

#### Vaccine allocation algorithms

The remaining vaccine doses in each year from 2020 to 2030 (black bars in Fig. 1) are then distributed according to one of three algorithms: (1) an optimised allocation by cervical cancer mortality caused by HPV 16 and 18 using the knapsack algorithm^25^, (2) a simple allocation by decreasing cervical cancer age-standardized mortality rate,^26^ and (3) an unoptimised allocation by decreasing human development index (HDI).^27^

The first (optimised) algorithm is a mathematical and computational approach that prioritises maximising the number of cervical cancer deaths averted. It uses the knapsack algorithm (see Supplementary Materials) to determine the dose allocation that maximises a given quantity (e.g., cervical cancer deaths averted) in the context of limited doses. We first calculated the ratio of cervical cancer deaths averted to vaccine demand for each pre-introduction country to rank the countries in order of greatest cancer death reduction per dose. We then used the knapsack algorithm to optimally allocate limited vaccine supply to maximise the vaccine benefits, i.e., cervical cancer deaths averted. Knapsack allows us to maximise one parameter (cervical cancer deaths averted) within the constraints of another parameter (vaccine dose required). Each country has a weight representing the number of vaccine doses needed and a value reflecting the potential number of cervical cancer deaths prevented by vaccinating that population. The total number of vaccine doses available for a particular year act as the knapsack's weight limit for that year. The goal is to maximise the total number of cervical cancer deaths prevented (value) by selecting the best combination of countries to vaccinate, all within the constraint of the available vaccines (knapsack capacity). In sensitivity analysis, we explored the impact of allocated the limited supply by cervical cancer cases averted.

A second (simple) algorithm allocates HPV vaccine doses to countries in the order of decreasing pre-vaccination cervical cancer mortality incidence.^26^ The algorithm prioritises countries based on their pre-vaccination cervical cancer mortality rate, allocating vaccines to countries with the highest number of cervical cancer deaths first. Given the available doses for a given year, we distribute doses to the country with the highest pre-vaccination mortality incidence. We then move down the list if there are available doses to be allocated and stop when the dose available is fewer than the dose required for the next country in line. The remaining countries that have yet to be selected are considered in the following year. This approach is easy to implement but does not explicitly consider factors like population size or the potential for future deaths.

A third (unoptimised) algorithm allocates supplies to countries in the order of decreasing HDI, using the 2019 HDI score obtained from the United Nations Development Programme.^27^ This reflects the historical situation where richer countries have generally been able to introduce HPV vaccination (and most other vaccines) ahead of poorer countries.^7^ Like the mortality incidence algorithm, we rank countries based on their HDI and allocate vaccine doses, starting with countries that have the highest HDI (mostly HICs) and moving down the list to those lowest HDI scores. Countries that are not selected in a particular year are considered in the following year. The algorithm allocates vaccines to countries with high HDI scores, allocating vaccines to more HICs first. Because the approach does not target the population most at risk for cervical cancer, it ignores countries that have a higher burden of cervical cancer. Hence this algorithm is unlikely to be optimal from a public health perspective standpoint, but may be a realistic model of the allocation produced by market forces alone.

All three algorithms are run until the year 2030, or when all countries are able to introduce HPV vaccines into their national programme, whichever is earlier. Data analyses were performed using the statistical software R.^28^

### Role of the funding source

The funders had no role in study design, data collection and analysis, decision to publish, or preparation of the manuscript. All authors (Kiesha Prem, Tania Cernuschi, Stefano Malvolti, Marc Brisson, Mark Jit) had full access to all of the study data and took final responsibility for the decision to submit for publication.

## Results

**Fig. 3:**
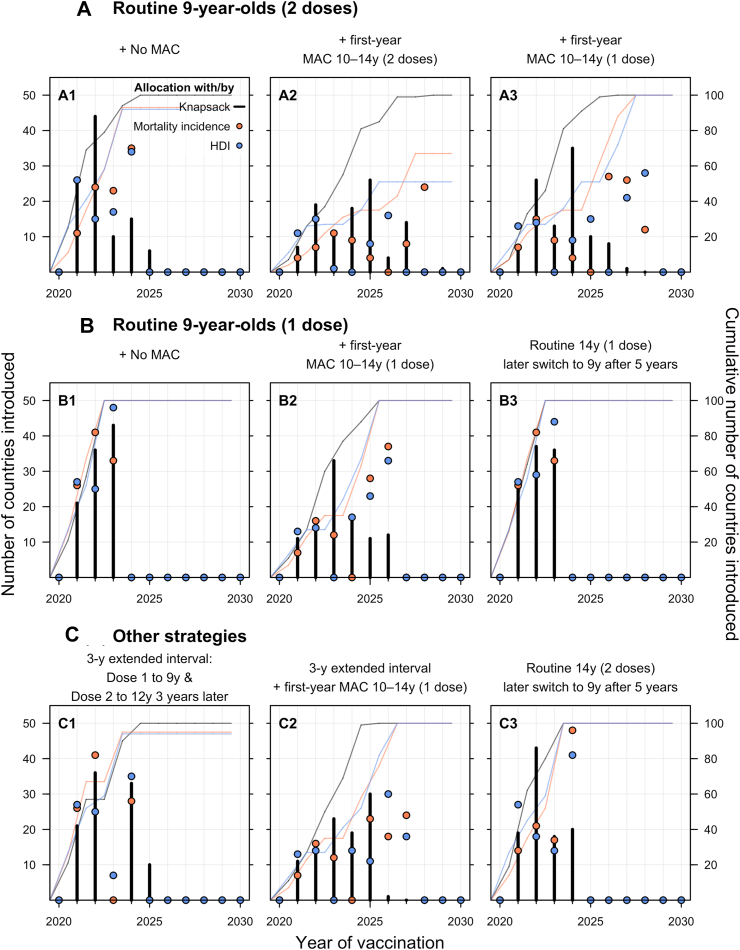
**Number of modelled HPV vaccine country introductions under different vaccination strategies over the years 2020**–**2030.** Allocation results from optimised and simple allocation by mortality and unoptimised allocation are presented as black lines and pink or blue points, respectively. The projected (and cumulative, as lines and on the secondary y-axis) number of countries that have been allocated to introduce routine HPV vaccination into their national programmes over the years 2020–2030. For detailed descriptions of the age cohorts vaccinated under the nine vaccination strategies, please refer to Fig. 2.

Strategies implementing MAC require more doses in the first year, resulting in a slower rollout of vaccination to all 100 countries compared to strategies without MAC or those that vaccinate older girls (14-year-olds) first. Routinely vaccinating girls aged 9 with one dose of the HPV vaccine (Strategy B1) is the strategy, across all the algorithms, that minimises the time needed for all the 100 countries to introduce HPV vaccination into their national immunisation programme (Fig. 3). The strategies that implemented routine vaccination of girls aged 9 with two doses with a first-year MAC up to age 14 (Strategy A2–3) were projected to take more time for global introduction.

**Fig. 4:**
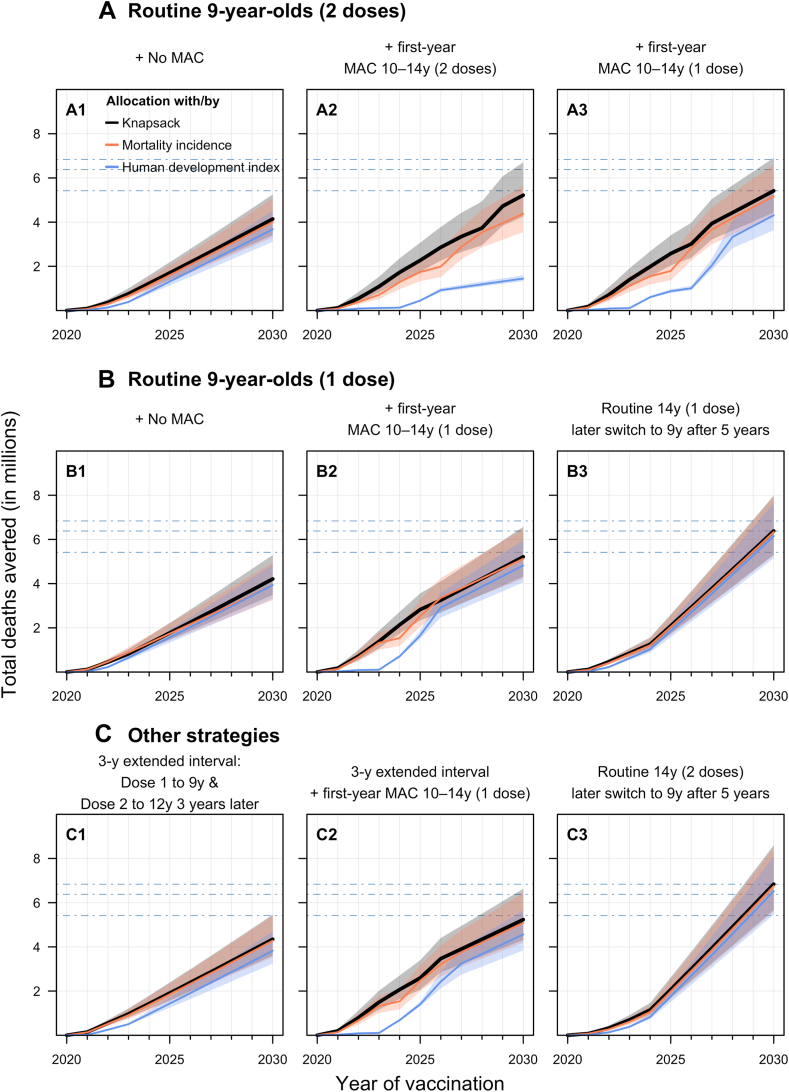
**Projected cumulative cervical cancer deaths averted from vaccination under different vaccination strategies over the years 2020**–**2030.** The projected cumulative number of cervical cancers averted because of vaccination for the different strategies over the years 2020–2030 are presented. Allocation results from optimised and simple allocation by mortality and unoptimised allocation are presented as black, pink or blue lines, respectively. The median is presented as lines and the shaded area is the 95% uncertainty intervals for each allocation. The dashed lines represent the projected total deaths averted by the top three most effective vaccination strategies (C3, B3, A3) identified in the optimised algorithm. For detailed descriptions of the age cohorts vaccinated under the nine vaccination strategies, please refer to Fig. 2.

**Fig. 5:**
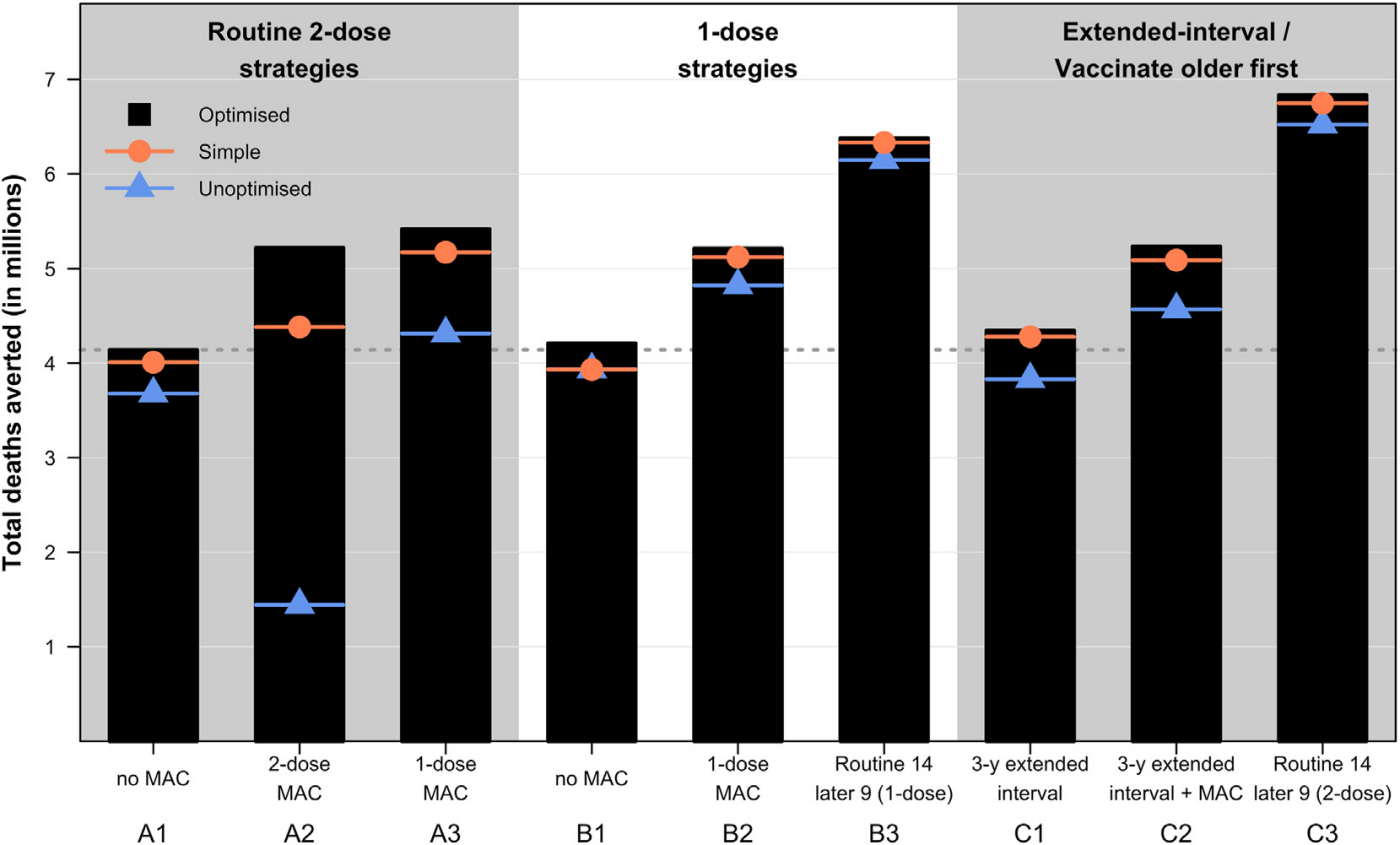
**Cumulative projected number of cervical cancers averted because of vaccination for the different strategies over the years 2020**–**2030.** The total projected number of cervical cancer deaths averted by the various vaccination strategies are presented. Allocation results from optimised and simple allocation by mortality and unoptimised allocation are presented as black bars, pink or blue lines/points, respectively. For detailed descriptions of the age cohorts vaccinated under the nine vaccination strategies, please refer to Fig. 2.

Across almost all vaccination strategies, the unoptimised allocation by HDI consistently performed worse, resulting in fewer new introductions (Fig. 3) and fewer cervical cancer deaths averted (Fig. 4) than allocation by cancer mortality incidence or knapsack (Fig. 5).

Routinely vaccinating 14-year-old girls with two doses and later switching to 9-year-olds (Strategy C3) is the strategy which, across all the algorithms, would avert the most cervical cancer deaths in the 100 countries (Figs. 4 and 5). Allocating the limited vaccine supplies by this strategy could avert around seven million cancer deaths among the individuals vaccinated in the years 2020–2030, with little difference across the country allocation algorithms. It remains the most effective strategy at preventing cancer deaths even under varying supplies even under potential HPV vaccine supply disruption from the COVID-19 pandemic (Supplementary Figures S2 and S3). Implementing the same vaccination strategy but with only one dose (Strategy B3) is the next best strategy at averting cervical cancer deaths in the 100 countries, regardless of allocation method (Fig. 5).

Routine two-dose or one-dose without MAC (Strategies A1 & B1) can avert about 4.1 million deaths and are among the least effective of the strategies considered at preventing cervical cancer deaths cause by HPV 16 and 18. Implementing MACs with one or two doses to 10–14-year-old girls in the first year (Strategies A2 & A3) can avert more than 5 million deaths (Fig. 5).

If one-dose dose confers lifelong protection at 90% vaccine take, the one-dose strategy of vaccinating 9-year-old girls routinely and implementing the first-year MAC with girls aged 10–14 (Strategy B2) prevents around 5.4 million cervical cancers averted among the individuals vaccinated in the years 2020–2030 (Fig. 5). Assuming lifelong protecting at 90% vaccine take, routine immunisation with one-dose and including a first-year MAC (Strategy B2) can also avert more than 1.2 million additional deaths than the two-dose routine-only schedule (Strategy A1). Assuming a higher vaccine efficacy of one dose (i.e., 95% instead of 90% with lifelong protection) results in one-dose strategies (Strategies B1–3) preventing more deaths, but still fewer than the strategy of routinely vaccinating 14-year-old girls with two doses and later switching to 9-year-olds (Strategy C3) (Supplementary Figures S5 and S6).

The 3-year extended-interval schedule (Strategy C1) may also prevent more deaths (4.2 million) compared to the routine-only schedules with one or two doses (Strategies A1 & B1). Moreover the 3-year extended interval schedule (Strategy C1) is also faster than the routine 2-dose without MAC (Strategy A1) when allocating limited vaccine supplies to the 100 countries (Fig. 3).

Similar results were observed if the optimised allocation algorithm (knapsack) and the simple allocation algorithm by decreasing mortality were designed to maximise cervical cancer cases averted instead of deaths (Supplementary Figure S7).

## Discussion

HPV vaccine supplies have been insufficient to meet demand over more than a decade, let alone the increased demand needed to reach WHO's cervical cancer elimination targets. The persistent lack of supply triggered several policy discussions at SAGE over the period 2019–2021. This study presents the modelling analysis conducted to inform SAGE discussions on allocating constrained vaccine supplies. The paper sheds light on the decision-making process behind some of the SAGE recommendations that helped to alleviate HPV supply constraints. With most existing production of vaccine doses taken up by countries with existing HPV immunisation programmes (>90% in 2019), a limited supply was left for country introductions, affecting many low and middle-income countries.^12^^,^^14^, ^15^, ^16^ The supply-constrained environment was exacerbated by COVID-19-related disruptions of manufacturing supply chains and delays in clinical development programmes.^19^^,^^20^ To optimise the use of scarce vaccine doses, we examined the impact of nine strategies for allocating doses within-country populations using three algorithms for allocating doses between countries. We found that if the vaccination strategy is efficient in preventing cervical cancer deaths, especially when supply is constrained, the order in which countries are allocated vaccines matters much less; all will maximise similar health gains. Vaccinating more age cohorts using dose-sparing strategies such as routinely vaccinating 14-year-old girls and switching to a routine 9-year-old programme (Strategy C3) could prevent the most (around seven million) cervical cancer deaths across all algorithms.

If the vaccination strategy is inefficient, then allocating vaccines to countries with higher HDI first will lead to fewer new introductions and deaths averted than simple and optimised allocation by mortality. While allocating doses to richer countries first is not the most impactful strategy, HPV vaccine rollout since licensure of the first vaccines in 2006 has followed this pattern due to the stronger purchasing power of rich countries as well as vaccine licensure being sought first in high-income markets (such as the United States and European Union). Failure to actively prioritise countries with the highest HPV burden, leaving prioritisation to be purely driven by market forces, can have a large cost in human lives. While recent improvements have been made in the global HPV vaccine supply situation, challenges persist. In 2024, reported manufacturing disruptions led to a shortfall of 10.7 million doses delivered by vaccine manufacturers to Gavi and UNICEF, disproportionately affecting low- and middle-income countries.^29^

The strategies with MAC (with one or two doses) or catch-ups with older girls (e.g., girls aged 14) prevent cervical cancers in cohorts of girls that would otherwise be missed in no-MAC strategies. However, strategies without MAC would allow for faster country introductions. We find that routinely vaccinating one cohort at age 9 without MACs does not perform very well compared to the other vaccination strategy evaluated. While a strategy with extended 3-year interval may not be optimal for averting cervical cancer deaths, implementing extended 5-year intervals could lead to more efficient use of the available supply.^30^

The strategy to vaccinate 14-year-olds with one or two doses until all countries have been able to introduce vaccination, then switching to 9-year-olds when supply is no longer constrained, is the most effective strategy (Strategy C3) in preventing cervical cancer deaths. Some countries may face practical challenges in achieving high vaccination coverage in 14-year-olds. In particular, many countries rely on school-based HPV vaccine delivery,^11^^,^^31^ and some of those countries, particularly the low and middle-income, have substantially lower female school enrolment rates in secondary school compared to primary school,^32^ potentially hindering high vaccination coverage among older adolescent girls. However, in Tanzania, vaccinating older girls has been demonstrated to be well-accepted^33^ and with high vaccine uptake.^34^ Moreover, further research is necessary to understand the long-term protection (beyond 20 years) of single-dose vaccination schedules.

Our analysis has several limitations in scope. We considered the optimal distribution of vaccines between and within countries but did not account for country-specific factors that might limit vaccine coverage even if supplies were available. Such factors include vaccine acceptance, affordability of procurement and distribution costs as well as programmatic feasibility of delivering vaccines. We also did not consider the preferences of countries for particular vaccines (e.g., based on valency or country of production). Moreover, as PRIME is a static model, it cannot estimate herd effects from vaccination, thus underestimating the true impact of vaccination in preventing cervical cancer deaths.^21^^,^^22^ Although we present a conservative outlook of the vaccine impact, not accounting for herd effects and cross-protection against non-vaccine HPV types, it is unlikely that the efficiency of the vaccination strategies in preventing cervical cancer deaths would differ much should transmission dynamics be considered.^35^^,^^36^

Implementing other interventions against cervical cancer, such as regular cervical screening, remains challenging in many low- and lower-middle-income countries and has not yielded a similar impact compared to high-income settings.^8^^,^^37^ In low-resource settings without well-established screening and treatment facilities, efficient allocation of limited HPV vaccine supplies is critical to eliminating cervical cancer.^2^

Policy decisions in conditions of scarce supply are very difficult and have undesired consequences: product allocations result in the roll-out of health interventions in certain populations at the expense of others. While unavoidable, it is paramount that those decisions are based on the best available evidence and the most accurate simulation of the impact of the alternative product allocation strategies. The case of the HPV vaccine, with its long-lasting shortage of supply, can represent a very effective case study to understand the impact that accurate modelling can have on policy decisions. To deal with the lack of supply, SAGE updated its guidance on HPV vaccination twice in 2019^16^ and 2022^18^; the results of the modelling efforts presented in this paper were one of the evidence sources used to inform those decisions. The result of these adjustments has led, together with the increase in manufacturing capacity and the success of various clinical development programs, to progressive ease of supply constraints,^38^ finally allowing countries still not having introduced the HPV vaccine in their schedule to plan for an introduction in the following years. The methodology developed in this paper remains valuable for future situations where vaccine shortages might arise due to unforeseen circumstances.

Vaccine supply shortages hamper the global immunisation effort to reduce the burden of vaccine-preventable diseases, disproportionately affecting the poorer economies when access to vaccines are left to market forces. As experienced during the current COVID-19 pandemic, vaccine allocations driven by pricing and nationalistic considerations can hinder efficient and equitable access to vaccines, with poorer economies at a disadvantage^19^^,^^39^^,^^40^ and causing avoidable increases in mortality and morbidity. By developing a modelling framework to project the health impact of HPV vaccination strategies achievable by optimally allocating limited vaccine supplies, we demonstrate the importance and impact of appropriate allocation frameworks informed by shared public health goals. Those frameworks can play a critical role in identifying the most efficient strategy in the context of vaccine supply constraints. Modelling may facilitate evidence-based decision-making that would allow scarce resources to be deployed more efficiently in the context of international collaboration towards enhanced health impact.

## Contributors

KP and MJ designed the study and led the overall data interpretation. TC, MB, and SM also participated in the study design. KP and MJ drafted the article. KP did the data analysis and model projections. KP, TC, and SM accessed and verified the data. All authors interpreted the results and critically revised the manuscript for scientific content. KP, TC, SM, MB, and MJ had full access to all the study data. All authors had the final responsibility to submit the manuscript for publication.

## Data sharing statement

All data used in this study can be downloaded from the cited references. The codes used to generate these analyses are available at https://github.com/kieshaprem/hpv_vaccine_supply.

## Editor note

The Lancet Group takes a neutral position with respect to territorial claims in published maps and institutional affiliations.

## Declaration of interests

All other authors involved in the research have confirmed that they had no commercial or financial interests that could potentially create a conflict of interest.

The funders had no role in study design, data collection and analysis, decision to publish, or preparation of the manuscript.
