# Supplementary material for: Optimal human papillomavirus vaccination strategies in the context of vaccine supply constraints in 100 countries
Source: eClinicalMedicine. 2024 Jul 18;74:102735. doi: 10.1016/j.eclinm.2024.102735 (PMC11293525; doi:10.1016/j.eclinm.2024.102735)
Supplement: Supplementary Materials [file mmc1.pdf]

Supplementary materials

Optimal human papillomavirus vaccination strategies in the context of vaccine supply constraints in 100 countries

Kiesha Prem[1], Tania Cernuschi, Stefano Malvolti, Marc Brisson, Mark Jit[1]

Table of Contents

Methods **2**
Vaccination strategies 3
Optimised allocation with knapsack 4
Results **5**
Reductions in supply projections due to the COVID-19 pandemic 5
Higher vaccine efficacy for one dose of the vaccine (i.e. 95%) 8
Maximising cervical cancer cases averted when allocating limited vaccine supplies 11

[1]Correspondence to kiesha.prem@lshtm.ac.uk and mark.jit@lshtm.ac.uk

Methods

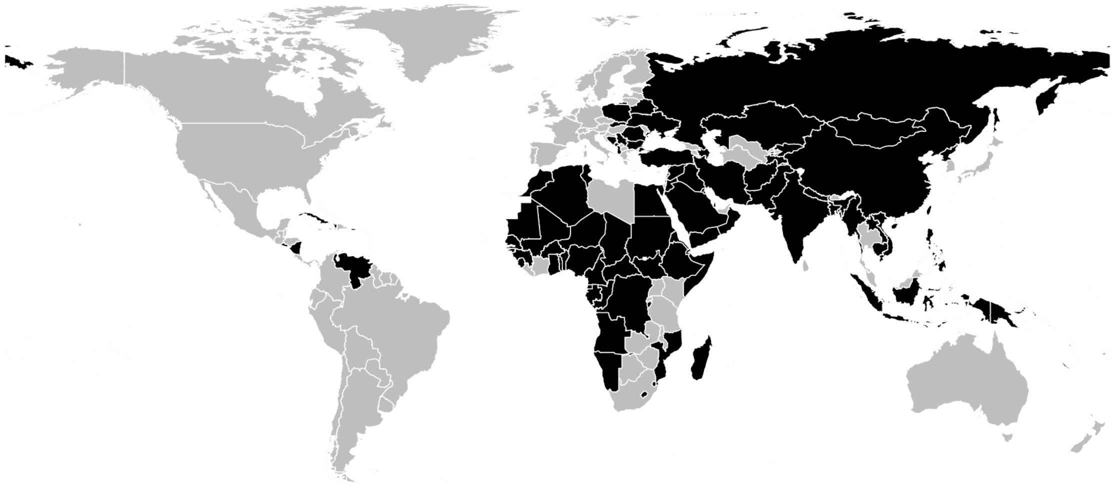

Supplementary Figure 1. Countries that have not introduced a national HPV immunisation programme in 2020. Of the 100 countries that have yet to introduce a national HPV immunisation programme, 60 are low- and lower-middle income countries and 24 are upper-middle-income countries. The French overseas departments—French Guiana, Guadeloupe, Martinique, and Reunion—are classified as high-income economies.

Vaccination strategies

The WHO Strategic Advisory Group of Experts on Immunization considered several vaccination strategies which allowed more countries to introduce vaccination despite constrained supplies. Routine HPV vaccination programmes target girls between 9 and 14 years of age. In the first year of HPV vaccine introduction, countries may also implement multiple age-cohort (MAC) vaccinations, vaccinating older girls that routine programmes would miss. Available vaccines were distributed according to one of nine vaccination strategies, which cover strategies with and without multiple age-cohort MAC or single-year catch-up, one- and two-dose and extended interval schedules, presented in **Supplementary Table 1**.

Supplementary Table 1. Nine vaccination strategies.

**Vaccination strategy**		**Description**
Routine 2 doses	Routine 2 doses no MAC[1]	Routine: 9-year-old girls are given 2 doses every year
	Routine 2 doses with MAC[1] (2 doses)	Routine: 9-year-old girls are given 2 doses every year<br>MAC[1]: 10–14-year-old girls are given 2 doses in the first year
	Routine 2 doses with MAC[1] (1 dose)	Routine: 9-year-old girls are given 2 doses every year<br>MAC[1]: 10–14-year-old girls are given 1 dose in the first year
Routine 1 dose	Routine 1 dose no MAC[1]	Routine: 9-year-old girls are given 1 dose every year
	Routine 1 dose with MAC[1] (1 dose)	Routine: 9-year-old girls are given 1 dose every year<br>MAC[1]: 10–14-year-old girls are given 1 dose in the first year
	Routine 14-year-old, later switch to routine 9-year-old (1-dose)	Routine: 14-year-old girls are given 1 dose for the first ten years. In the sixth year when supply is no longer constrained, 9-year-old girls are given 1 dose every year
Extended interval/vaccinating older girls	3-year extended interval	Routine: 9-year-old girls are given their first dose<br>Extended interval: Three years later, the girls (now aged 12) are given their second dose
	3-year extended interval + MAC[1] (1-dose)	Routine: 9-year-old girls are given their first dose<br>MAC[1]: 10–14-year-old girls are given 1 dose in the first year<br>Extended interval: Three years later, the girls (now aged 12) are given their second dose
	Routine 14-year-old, later switch to routine 9-year-old	Routine: 14-year-old girls are given 2 doses for the first ten years. In the sixth year when supply is no longer constrained, 9-year-old girls are given 2 doses every year

[1]MAC: multiple age-cohort vaccinations

Optimised allocation with knapsack

To optimise the use of scarce vaccine doses, we examined the health impact of using knapsack for allocating doses between pre-introduction countries and nine vaccination strategies for allocating doses within-country populations. Here, we describe the knapsack algorithm adopted.

Which countries should be selected for country introduction in a particular year? Over the years 2020 to 2030, we want to allocate limited vaccine supplies to pre-introduction countries for their HPV vaccination programme. As of May 2020, 100 such countries have yet to introduce a national HPV immunisation programme (**Supplementary Figure 1**), and the vaccine demand of country c is w_i^c for year i. If country c introduces HPV vaccination in year i, it could prevent p_i^c cervical cancer deaths (or cases); the profit of selecting country c. We assume that once countries have introduced HPV into their immunisation programme, they will implement an annual routine vaccination programme. To simplify the problem, we do not consider partial introductions, vaccine acceptance or preferences (e.g., based on valency or country of production), affordability of procurement and distribution costs, or programmatic feasibility of delivering vaccines.

Given the following parameters,
v_i = the annual capacity, i.e., the number of vaccine doses available in year i,
N_i = the number of pre-introduction countries in year i,
w_i^c = the weight, vaccine demand of each country c in year i, for $c = 1, 2, \ldots, N_i$,
p_i^c = the value associated with country c in year i, i.e., the deaths (or cases) that can be averted if country c introduces in year i and routinely vaccinated from year i onwards, for $c = 1, 2, \ldots, N_i$.

Then, we formulate our 0-1 knapsack problem as:
Maximise

$$\sum p_i^c x_i^c$$

Subject to:

$$\sum w_i^c x_i^c \leq v_i,$$

where $x_i^1, x_i^2, \ldots, x_i^N$ take values 0 or 1, such that if $x_i^c = 1$, then county c is selected for introduction in year i. We solve this single knapsack problem by using the dynamic programming approach in R (R Foundation for Statistical Computing). We performed optimised allocation (knapsack) for all nine vaccination strategies under different supply projections (5% reduction as base case, and 0% and 10% reduction as sensitivity analyses).

Results

Reductions in supply projections due to the COVID-19 pandemic

If doses were reduced by 10% (instead of 5%) due to the COVID-19 pandemic, then about half of the countries would be unable to introduce vaccination before 2030 in routine two-dose with MAC when limited supplies were allocated by cancer mortality or HDI (**Supplementary Figure 2**). Only the knapsack algorithm found a solution to ensure that all 100 countries could introduce HPV vaccination before 2030 across all strategies, even when supplies were further reduced because of COVID-19. However, routinely vaccinating girls aged 9 with two doses without MAC remains the most efficient strategy if vaccine supplies were reduced by 10% or if they were unchanged (**Supplementary Figures 2 and 3**).

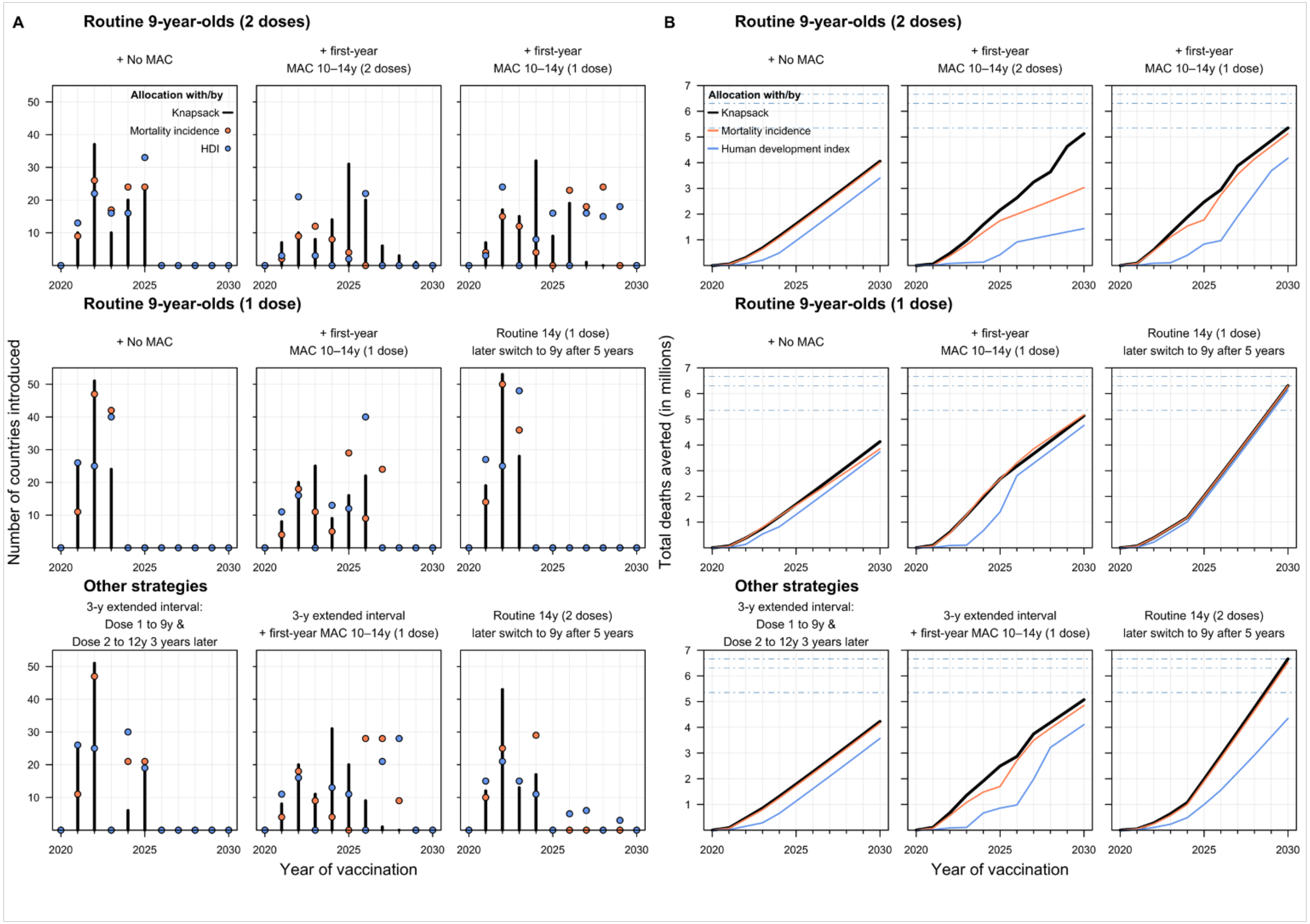

Supplementary Figure 2. Number of HPV vaccine country introductions and cervical cancer deaths averted from vaccination under different vaccination strategies over the years 2020–2030 with 10% vaccine supply reduction. The projected number of countries that have been allocated to introduce routine HPV vaccination into their national programmes over the years 2020–2030 (panel A). The projected number of cervical cancer deaths averted because of vaccination for the different strategies over the years 2020–2030 are presented in panel B. In this sensitivity analysis, there is a 10% reduction in vaccine supply.

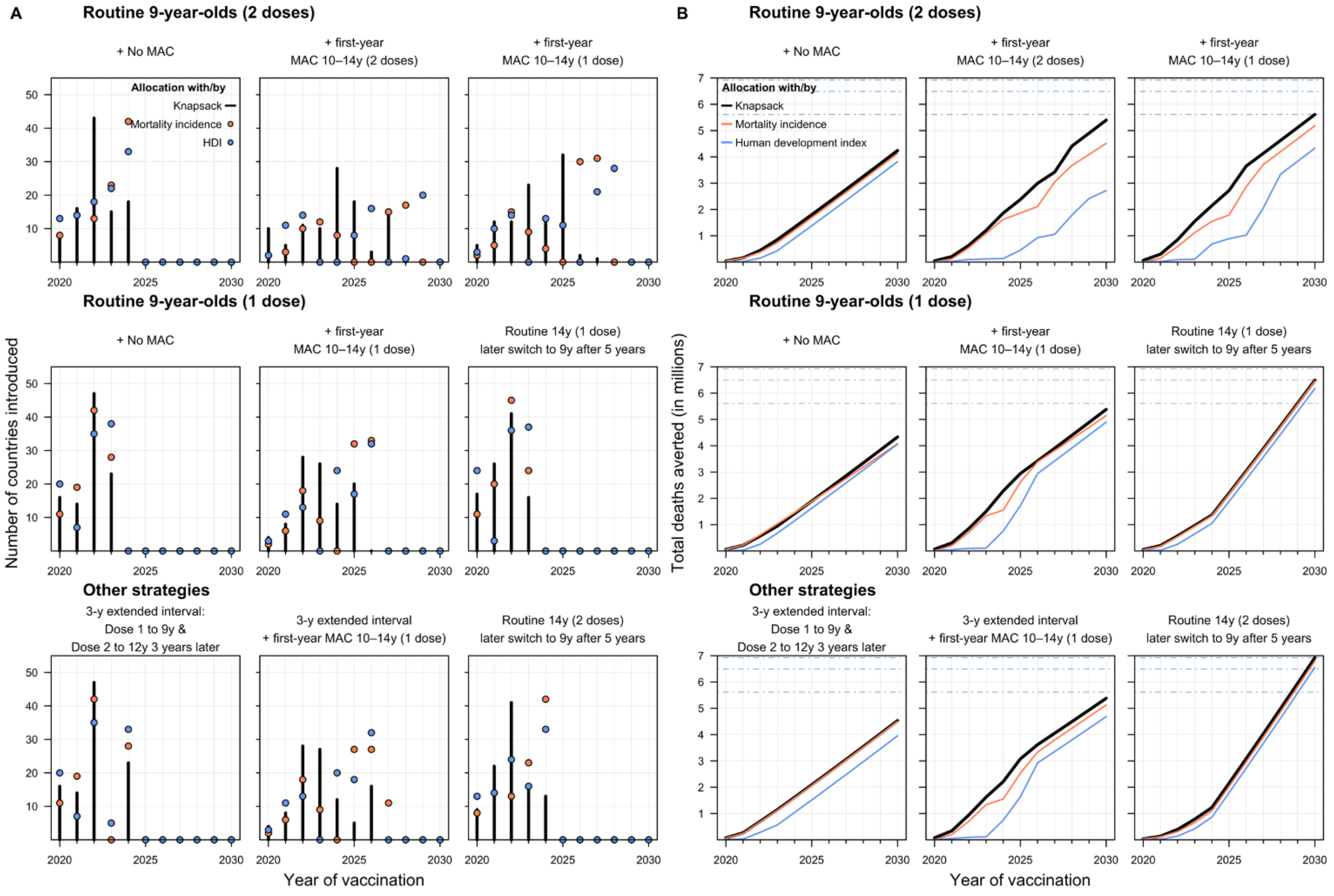

Supplementary Figure 3. Number of HPV vaccine country introductions and cervical cancer deaths averted from vaccination under different vaccination strategies over the years 2020–2030 with no vaccine supply reduction. The projected number of countries that have been allocated to introduce routine HPV vaccination into their national programmes over the years 2020–2030 (panel A). The projected number of cervical cancer deaths averted because of vaccination for the different strategies over the years 2020–2030 are presented in panel B. In this sensitivity analysis, there is no reduction in vaccine supply.

Higher vaccine efficacy for one dose of the vaccine (i.e. 95%)

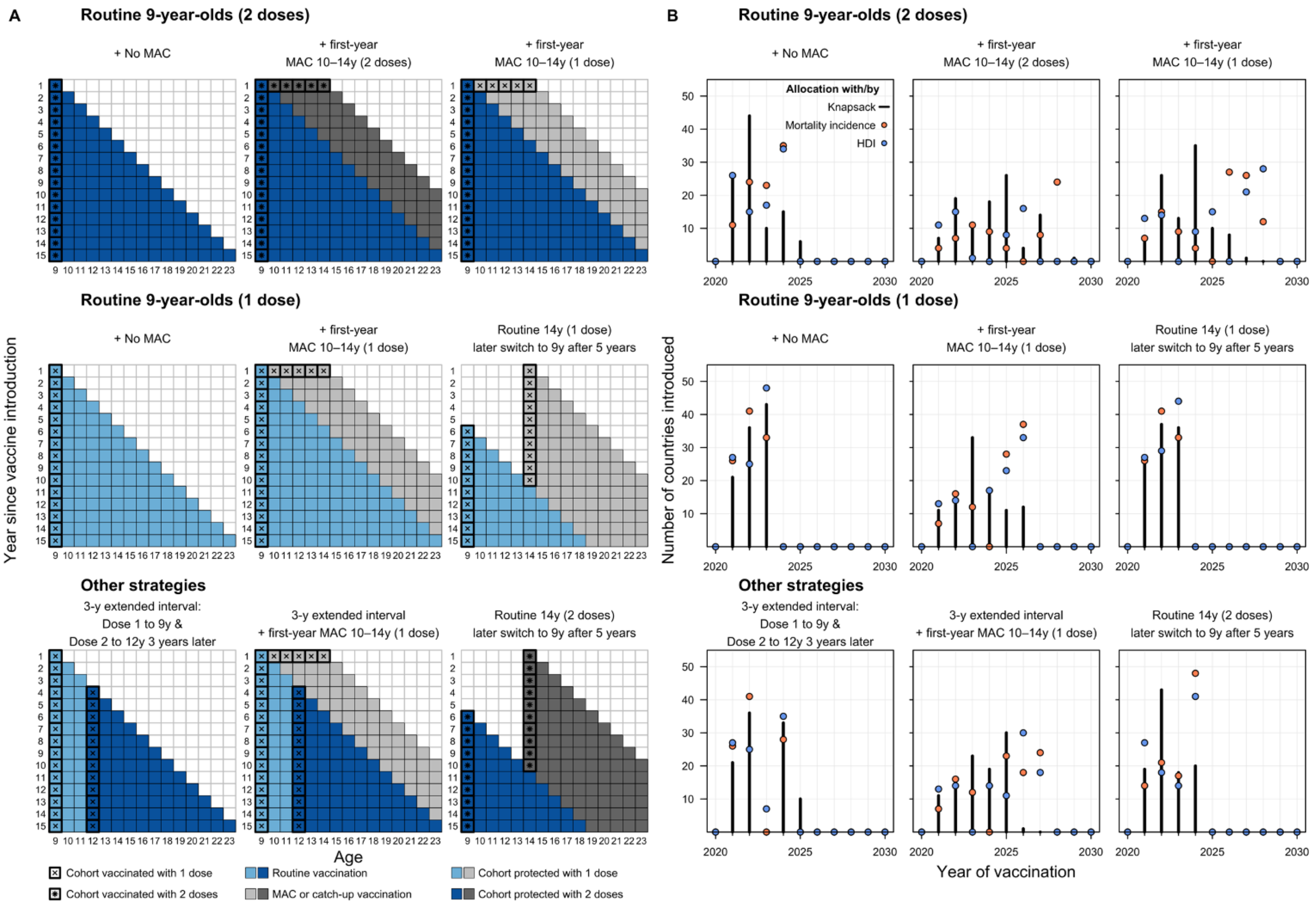

Supplementary Figure 4. Number of modelled HPV vaccine country introductions under different vaccination strategies over the years 2020–2030. The age cohorts vaccinated under the nine vaccination strategies considered are presented (in panel A). Allocation results from optimised and simple allocation by mortality and unoptimised allocation are presented as black lines and pink or blue points, respectively. The projected number of countries that have been allocated to introduce routine HPV vaccination into their national programmes over the years 2020–2030 (in panel B).

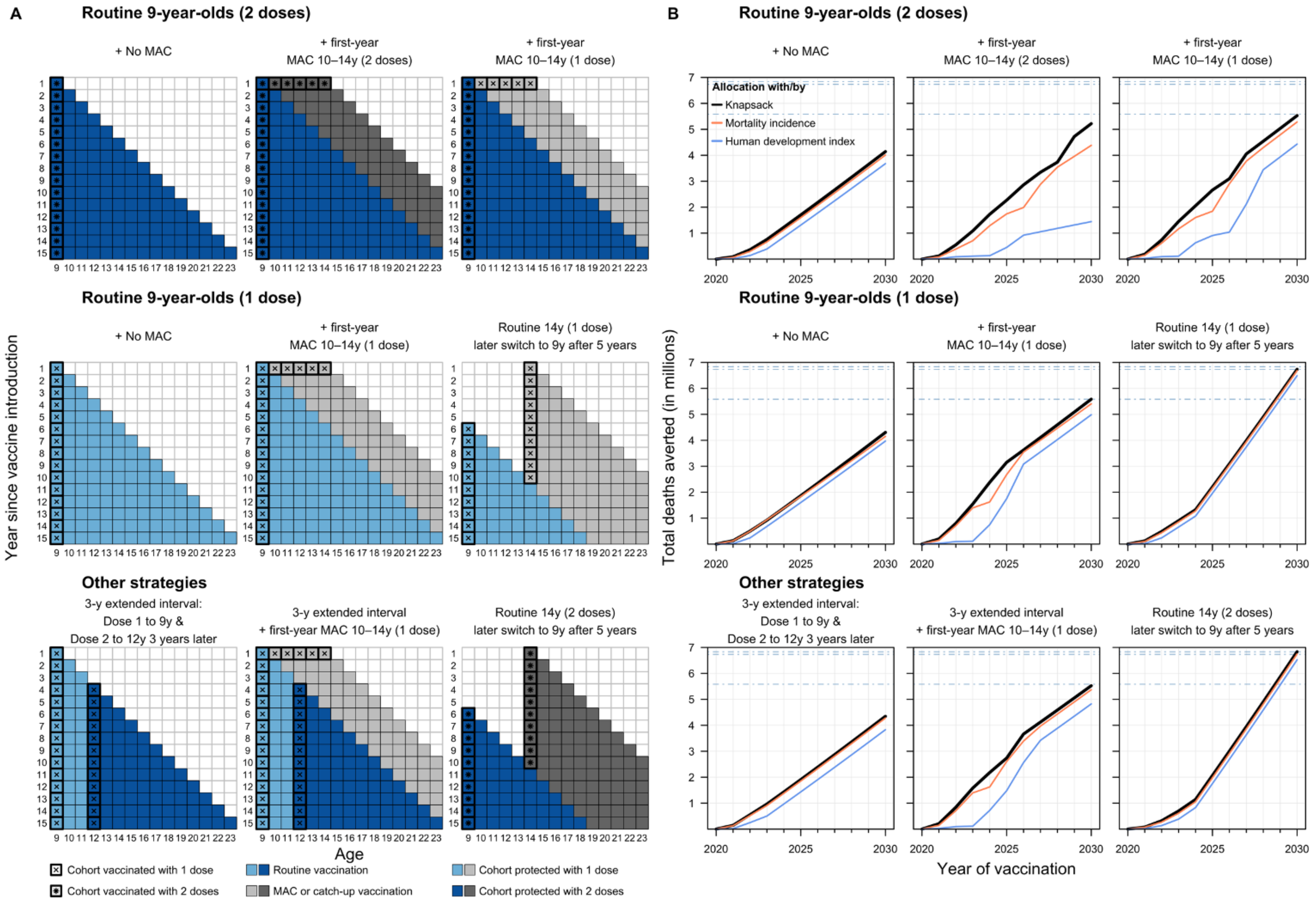

Supplementary Figure 5. Projected cumulative cervical cancer deaths averted from vaccination under different vaccination strategies over the years 2020–2030. The age cohorts vaccinated under the nine vaccination strategies considered are presented (in panel A). Allocation results from optimised and simple allocation by mortality and unoptimised allocation are presented as black, pink or blue lines, respectively. The projected number of countries that have been allocated to introduce routine HPV vaccination into their national programmes over the years 2020–2030 (in panel B). The projected number of cervical cancers averted because of vaccination for the different strategies over the years 2020–2030 are presented in panel B.

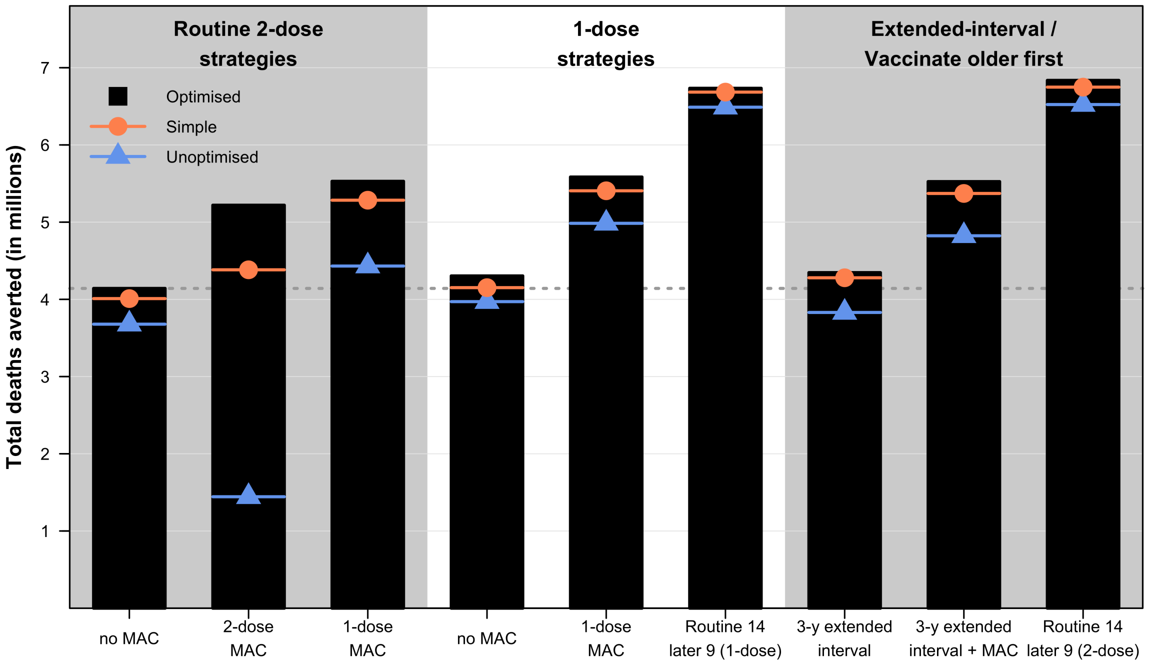

Supplementary Figure 6. Cumulative projected number of cervical cancers averted because of vaccination for the different strategies over the years 2020–2030. The total projected number of cervical cancer deaths averted by the various vaccination strategies are presented. Allocation results from optimised and simple allocation by mortality and unoptimised allocation are presented as black bars, pink or blue lines/points, respectively.

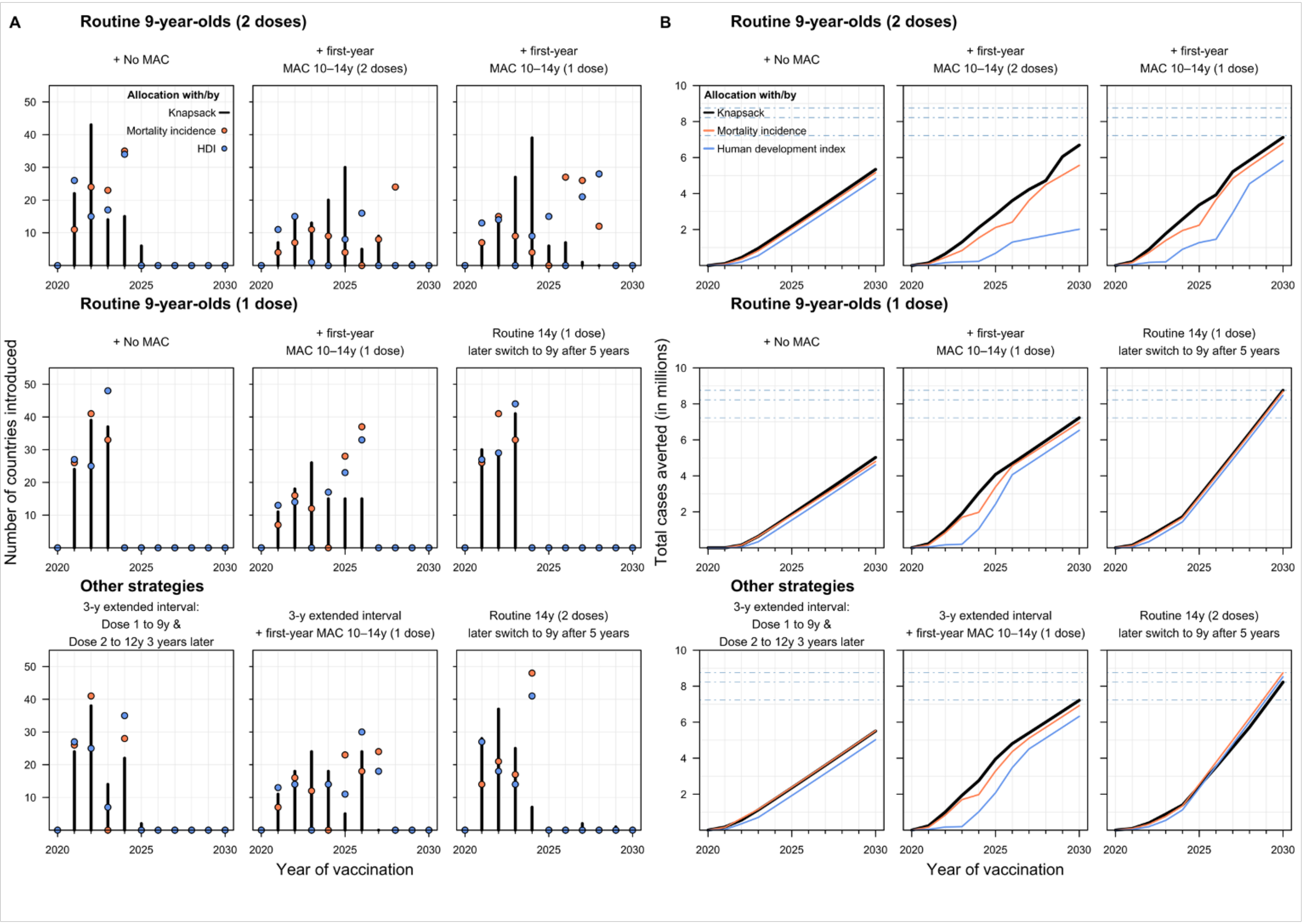

Supplementary Figure 7. Number of HPV vaccine country introductions and cervical cancer cases averted from vaccination under different vaccination strategies over the years 2020–2030 when allocating supplies to maximise cancers averted. The projected number of countries that have been allocated to introduce routine HPV vaccination into their national programmes over the years 2020–2030 (panel A). The projected number of cervical cancer deaths averted because of vaccination for the different strategies over the years 2020–2030 are presented in panel B.

When this analysis was initially conducted to inform WHO recommendations, it centred on the efficacy of one dose due to evidence from trials and post-randomisation analyses indicating consistent antibody titres(1,2). The focus was directed towards understanding the vaccine take (initial efficacy) of a single dose. Findings from the KENSHE trial(3) revealed that the vaccine uptake for a single dose against incident persistent oncogenic HPV infection may be high at 95%, demonstrating comparable effectiveness to a multi-dose regimen, alleviating uncertainties around the efficacy of one-dose regimens. The current attention has thus shifted to assessing the duration of protection conferred by a single dose. Vaccination strategies administering the second dose may become more effective if the duration of protection conferred by one dose misses the population's peak sexual activity (4,5).

Reference

1. Porras C, Sampson JN, Herrero R, Gail MH, Cortés B, Hildesheim A, et al. Rationale and design of a double-blind randomized non-inferiority clinical trial to evaluate one or two doses of vaccine against human papillomavirus including an epidemiologic survey to estimate vaccine efficacy: The Costa Rica ESCUDDO trial. Vaccine. 2022 Jan 3;40(1):76–88.

2. Basu P, Malvi SG, Joshi S, Bhatla N, Muwonge R, Lucas E, et al. Vaccine efficacy against persistent human papillomavirus (HPV) 16/18 infection at 10 years after one, two, and three doses of quadrivalent HPV vaccine in girls in India: a multicentre, prospective, cohort study. The Lancet Oncology. 2021 Nov 1;22(11):1518–29.

3. Barnabas RV, Brown ER, Onono MA, Bukusi EA, Njoroge B, Winer RL, et al. Efficacy of Single-Dose Human Papillomavirus Vaccination among Young African Women. NEJM Evidence [Internet]. 2022 Apr 11 [cited 2022 Jun 20];1(5). Available from: https://evidence.nejm.org/doi/abs/10.1056/EVIDoa2100056

4. Drolet M, Laprise JF, Martin D, Jit M, Bénard É, Gingras G, et al. Optimal human papillomavirus (HPV) vaccination strategies to prevent cervical cancer in low- and middle-income countries in the context of limited resources: A mathematical modeling analysis. Lancet Infectious Diseases. 2021;

5. Bénard É, Drolet M, Laprise JF, Gingras G, Jit M, Boily MC, et al. Potential population-level effectiveness of one-dose HPV vaccination in low-income and middle-income countries: a mathematical modelling analysis. The Lancet Public Health. 2023 Oct;8(10):e788–99.
